# A Review on Early Forest Fire Detection Systems Using Optical Remote Sensing

**DOI:** 10.3390/s20226442

**Published:** 2020-11-11

**Authors:** Panagiotis Barmpoutis, Periklis Papaioannou, Kosmas Dimitropoulos, Nikos Grammalidis

**Affiliations:** Centre for Research and Technology Hellas, Information Technologies Institute, 57001 Thessaloniki, Greece; panbar@iti.gr (P.B.); ppapaioa@iti.gr (P.P.); dimitrop@iti.gr (K.D.)

**Keywords:** early fire detection, multispectral imaging systems, terrestrial, aerial, satellite, artificial intelligence

## Abstract

The environmental challenges the world faces nowadays have never been greater or more complex. Global areas covered by forests and urban woodlands are threatened by natural disasters that have increased dramatically during the last decades, in terms of both frequency and magnitude. Large-scale forest fires are one of the most harmful natural hazards affecting climate change and life around the world. Thus, to minimize their impacts on people and nature, the adoption of well-planned and closely coordinated effective prevention, early warning, and response approaches are necessary. This paper presents an overview of the optical remote sensing technologies used in early fire warning systems and provides an extensive survey on both flame and smoke detection algorithms employed by each technology. Three types of systems are identified, namely terrestrial, airborne, and spaceborne-based systems, while various models aiming to detect fire occurrences with high accuracy in challenging environments are studied. Finally, the strengths and weaknesses of fire detection systems based on optical remote sensing are discussed aiming to contribute to future research projects for the development of early warning fire systems.

## 1. Introduction

Over the last few years, climate change and human-caused factors have a significant impact on the environment. Some of these events include heat waves, droughts, dust storms, floods, hurricanes, and wildfires. Wildfires have extreme consequences on local and global ecosystems and cause serious damages to infrastructure, injuries, and losses in human lives; therefore, fire detection and the accurate monitoring of the disturbance type, size, and impact over large areas is becoming increasingly important [1]. To this end, strong efforts have been made to avoid or mitigate such consequences by early fire detection or fire risk mapping [2]. Traditionally, forest fires were mainly detected by human observation from fire lookout towers and involved only primitive tools, such as the Osborne fire Finder [3]; however, this approach is inefficient, as it is prone to human error and fatigue. On the other hand, conventional sensors for the detection of heat, smoke, flame, and gas typically take time for the particles to reach the point of sensors and activate them. In addition, the range of such sensors is relatively small, hence, a large number of sensors need to be installed to cover large areas [4].

**Figure 1:**
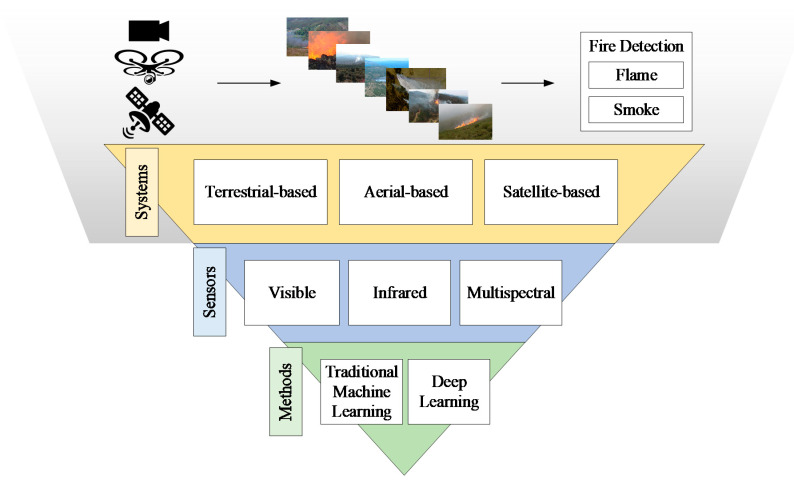
Generalized multispectral imaging systems for early fire detection.

**Figure 2:**
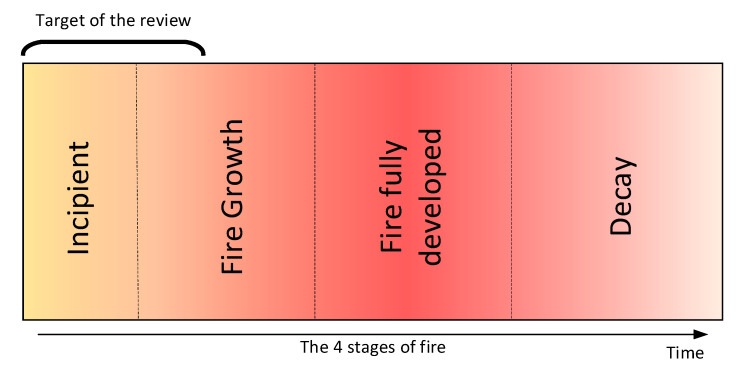
Systems discussed in this review target the detection of fire in the early stages of the fire cycle.

Recent advances in computer vision, machine learning, and remote sensing technologies offer new tools for detecting and monitoring forest fires, while the development of new materials and microelectronics have allowed sensors to be more efficient in identifying active forest fires. Unlike other fire detection review papers that have focused on various sensing technologies [5], on video flame or/and smoke methodologies in visible or/and InfraRed (IR) range [6,7,8,9], on various environments [10], and airborne systems [11,12], in this paper, we provide a comprehensive study of the most representative forest fire detection systems, focusing on those that use optical remote sensing, as well as digital image processing [13] and classification techniques [14]. Depending on the acquisition level, three broad categories of widely used systems that can detect or monitor active fire or smoke incidents in real/near-real-time are identified and discussed, namely terrestrial, aerial, and satellite. These systems are usually equipped with visible, IR, or multispectral sensors whose data are processed by machine learning methods. These methods rely either on the extraction of handcrafted features or on powerful deep learning networks (Figure 1) for early detection of forest fires (Figure 2) as well as for modeling fire or smoke behavior. Finally, we present the strengths and weaknesses of the aforementioned methods and sensors, as well as future trends in the field of early fire detection.

This paper is organized as follows: Section 2 covers different optical remote sensing systems for early fire detection, organized into three subsections—for Terrestrial, Aerial, and Satellite systems respectively—Section 3 includes discussion and the future scope of research. 

## 2. Early Fire Detection Systems

### 2.1. Terrestrial Systems

Terrestrial-based early detection systems consist of either individual sensors (fixed, PTZ, or 360° cameras) or networks of ground sensors. These sensors need to be carefully placed to ensure adequate visibility. Thus, they are usually located in watchtowers, which are structures located on high vantage points for monitoring high-risk situations and can be used not only for detection but also for verification and localization of reported fires. There are two types of cameras used for early fire detection, namely optical cameras and IR cameras that can capture data ranging from low resolution to ultra-high resolution for different fire detection scenarios [15]. Optical cameras provide color information, whereas IR imaging sensors can provide a measure of the thermal radiation emitted by objects in the scene [16]. More recently, early detection systems that combine both types have also been introduced. The computer-based methods can process a high number of data aiming to achieve a consistent level of accuracy maintaining a low false alarm rate. In the following, we first present traditional approaches that are based on handcrafted features followed by more recent methods using deep learning for automated feature extraction.

#### 2.1.1. Traditional Methods

Detection methods that use optical sensors or RGB cameras combine features that are related to the physical properties of flame and smoke, such as color, motion, spectral, spatial, temporal, and texture characteristics. The following color spaces have been used for the task of early fire detection: RGB [17,18,19], YCbCr [20], CIELAB [21], YUV [22,23], and HSV [24]; however, a drawback of color-based fire detection models is the high false alarm rates, since single-color information is insufficient in most cases for the early and robust fire detection. Thus, many of the developed methodologies combine color and motion information in images and videos [25]. Zhang et al. [26] used a probabilistic color-based model for the detection of fire regions and motion features for the final fire existence occurrence decision. Avgerinakis et al. [27] identified the smoke candidate blocks and then constructed the histograms of oriented gradients (HOGs) and histograms of optical flow (HOFs), thus taking into account both appearance and motion information. Likewise, Mueller et al. [28] used two optical flow schemes, namely optimal mass transport models and data-driven optical flow models.

Other researchers focused on the flickering effect of fire. This is observed in flame contours at a frequency of around 10 Hz, independently of the burning material and the burner [29]. To this end, Gunay et al. [30] distinguished flame flicker from the motion of fire-colored moving objects in videos using hidden Markov models (HMMs). Training HMMs leads to the reduction of data redundancy and improvement of reliability, while real-time detection is also achieved [31]. Furthermore, HMM-based methods for fire detection in the compressed domain have been proposed in MJPEG2000 [32] and H. 264 [33] compressed video. The use of multi-feature fire-based detection can offer more accurate results. Chen et al. [34] combined motion detection using a Gaussian mixture model, color analysis using an RGB color filtering, and flickering temporal analysis. The algorithm was applied to a video dataset consisting of different daytime and nighttime environments; however, at night, color analysis is less useful and night smoke is less visible. Thus, nighttime wildfire detection typically relies on motion analysis. Also, Töreyin, et al. [35] proposed a system equipped with an optical camera and a methodology that combines feature extraction (moving pixel/region extraction, color-based segmentation, and wavelet analysis in temporal and spatial domains), followed by a voting-based classifier. In [36], Barmpoutis et al. extracted an additional feature aiming to estimate a spatio–temporal consistency energy. Thereafter, they used a support vector machine (SVM) classifier to increase the robustness of fire detection.

Many other researchers have used infrared cameras aiming to reduce the false alarm rates of the optical-based terrestrial systems. The IR cameras measure the thermal radiation emitted by objects within the spectral range from either 3 to 5 μm (middle wavelength infrared, MWIR) or 8 to 14 μm (long wavelength infrared, LWIR). The MWIR band detectors, although they are optimal for fire detection, are expensive due to the cooling system required, so typically LWIR cameras are used. In IR videos the existence of rapid time-varying contours is an important sign of the presence of fire in the scene. Arrue et al. [37] observed that fire detection systems fail for distant fires and proposed a system for false alarm reduction in infrared forest-fire detection. More specifically, they used an adaptive infrared threshold, a segmentation method, and a neural network for early fire detection. Furthermore, Töreyin, et al. [16] used IR video to overcome the limitations of optical cameras in the detection of fires with little radiance in the visible spectrum. Specifically, they first estimated the boundaries of moving bright regions in each frame and then used spatio–temporal analysis in the wavelet domain using HMMs.

In contrast to single-sensor systems, multisensor systems typically cover wider areas and can achieve higher accuracies by fusing data from different sensors. The idea of integrated early fire detection and monitoring by combining data from optical and infrared cameras as well as a wireless sensor network (WSN) of temperature/humidity sensors was proposed by Grammalidis et al. [38]. Sensor data were processed and transmitted to a monitoring center employing computer vision and pattern recognition algorithms for automated fire detection and localization. The algorithm took into account color, spatial, and temporal information for flame detection, while for smoke detection, an online adaptive decision fusion (ADF) framework was developed. This framework consisted of several algorithms aiming to detect slow-moving objects, smoke-colored regions, and smoke region smoothness. Furthermore, improved early wildfire detection was achieved by fusing smoke detection from visual cameras and flame detection from infrared (LWIR) cameras. Similarly, Bosch et al. [39] proposed a system consisting of a wireless sensor network with a central monitoring station. In this, each sensor consists of an optical and a thermal camera and an integrated system for the processing of data and communication.

More recently, Barmpoutis et al. [40] and Dimitropoulos et al. [41] introduced fire detection systems based on smoke and flame dynamic texture analysis through linear dynamical systems (LDSs). Their modeling, combining color, motion, and spatio–temporal features led to higher detection rates and a significant reduction of false alarms. Temporal and spatial dynamic texture analysis of flame for forest fire detection was performed in [42]. Dynamic texture features were derived using two-dimensional (2D) spatial wavelet decomposition in the temporal domain and three-dimensional (3D) volumetric wavelet decomposition. In [43], the authors improved the smoke modeling of the fire incidents through dynamic textures solving higher order LDS (h-LDS). Finally, in [44], the authors took the advantage of the geometric properties of stabilized h-LDS (sh-LDS) space, and they proposed a novel descriptor, namely, histograms of Grassmannian points (HoGP) to improve the classification of both flame and smoke sequences.

#### 2.1.2. Deep Learning Methods

In contrast to previously discussed methods that rely on handcrafted features, deep learning (DL) methods [45] can automatically extract and learn complex feature representations. Since the seminal work of Krizhevsky et al. [46], which achieved exceptional image classification performance by training a convolutional neural network (CNN) with efficient computation resources, deep learning is one of the most rapidly evolving fields and has been successfully applied to numerous computer vision problems. To this end, Luo et al. [47] developed a smoke detection algorithm based on the motion characteristics of smoke and a CNN. Firstly, they identified the candidate regions based on the background dynamic update and dark channel a priori method [48]. Then, the features of the candidate region were extracted automatically by a CNN consisting of five convolutional layers and three fully connected layers. In [49], the authors combined deep learning and handcrafted features to recognize the fire and smoke areas. For static features, the AlexNet architecture was adapted, while for dynamic features an adaptive weighted direction algorithm was used. Moreover, Sharma et al. [50] used optical images and re-tuned two pre-trained CNNs, based on VGG16 and ResNet50 backbones to distinguish images that contain fire and images that do not. It is worth mentioning that for the training, they created an unbalanced dataset including more non-fire images. Zhang et al. [51] proposed deep CNNs for forest fire detection in a cascaded fashion. Firstly, they trained a full image fire classifier to decide whether the image contains the fire or not and then applied a fine-grained patch classifier to localize the fire patches within this image. The full image classifier is a deep CNN that has been fine-tuned from AlexNet and the fine-grained patch classifier is a two-layer fully connected neural networks trained with the upsampled Pool-5 features. Muhammad et al. [52], inspired by GoogleNet architecture and proposed a fine-tuned fire detection CNN model for surveillance videos, while Shen et al. [53] used an optimized YOLO model for flame detection from video frames. Frizzi et al. [54] built a simple CNN consisting of nine layers. The architecture of this model was similar to LeNet-5 including dropout layers and used a leaky rectified linear unit (ReLU) activation function. Muhamad et al. [55] proposed a fine-tuned CNNs architecture based on the SqueezeNet model and developed a feature map selection algorithm for fire segmentation and background analysis. In [56], the authors combined AlexNet as a baseline architecture and the internet of multimedia things (IoMT) for fire detection and disaster management. The developed system introduced an adaptive prioritization mechanism for cameras in the surveillance system allowing high-resolution cameras to be activated to confirm the fire and analyze the data in real-time. Furthermore, Dunnings and Breckon [57] used low-complexity CNN architectural variants and applied a superpixel localization approach aiming to reduce the computational performance offering up to 17 fps processing time.

Since the number of publicly available wildfire datasets is still limited, Sousa et al. [58] proposed a fire detection method based on data augmentation and transfer learning. A method that had been pre-trained on the ImageNet Inception-v3 model was retrained and evaluated using ten-fold cross-validation on the Corsican Fire Database [59]. Extending deep learning approaches, Barmpoutis et al. [60] combined the power of faster region-based convolutional neural network (R-CNN) with multidimensional dynamic texture analysis based on higher-order LDSs aiming the early forest fire detection. A modified faster R-CNN and a 3D CNN were combined in [61]. To that end, the faster R-CNN with non-maximum annexation was utilized to realize the smoke target location based on static spatial information and then a 3D CNN was used for smoke recognition by combining dynamic spatial–temporal information. Jadon et al. [62] developed the FireNet convolution neural network using a standard fire dataset and a self-proposed dataset, achieving an encouraging performance in terms of a series of evaluation metrics. Zhang et al. [63] trained a faster R-CNN for forest smoke detection by creating synthetic smoke images and achieved great performance when tested on real smoke images. Moreover, in [64] the authors extracted spatial features through a faster R-CNN for the detection of the suspected regions of fire (SroFs) and non-fire. Then, the features of the detected SroFs in successive frames were used by a long short-term memory (LSTM) for them to identify whether there is a fire or not in a short-term period. Finally, a majority voting method and the exploitation of fire dynamics were used for the final decision. Shi et al. [65] inspired by the idea of R-CNN and combined image saliency detection and convolutional neural networks. More specifically, they utilized the pixel-wise image saliency aggregating (PISA) method [66] to identify the candidate regions and then classified them into fire or non-fire regions.

Instead of extracting bounding boxes, Yuan et al. [67] used a two-path encoder-decoder fully convolutional network (FCN) for visual smoke segmentation. FCNs can achieve end-to-end pixel-wise segmentation so the precise location of smoke can be identified in images. They also created synthetic smoke images instead of labeling the real smoke images manually for training and then tested the network on both synthetic and real videos. Cheng et al. [68] proposed a smoke detection model using Deeplabv3+ and a generative adversarial network (GAN). The smoke pixels were first identified by fusing the result Deeplabv3+ and the heatmap of smoke based on HSV features. Then, a GAN was employed for predicting the smoke trend heatmap based on the space–time analysis of the smoke videos. Finally, in [69] the authors used a two-stage training of deep convolutional GANs for smoke detection. This procedure included a regular training step of a deep convolutional (DC)-GAN with real images and noise vectors and a training step of the discriminator separately using the smoke images without the generator.

### 2.2. Unmanned Aerial Vehicles

Terrestrial imaging systems can detect both flame and smoke, but in many cases, it is almost impossible to view, in a timely manner, the flames of a wildfire from a ground-based camera or a mounted camera on a forest watchtower. To this end, autonomous unmanned aerial vehicles (UAVs) can provide a broader and more accurate perception of the fire from above, even in areas that are inaccessible or considered too dangerous for operations by firefighting crews. Either fixed or rotary-wing UAVs cover wider areas and are flexible, allowing the change of monitoring area, but they are affected by weather conditions and have limited flight time. UAVs mostly use ultra-high-resolution optical or infrared charge-coupled device (CCD) cameras to capture images as well as other sensors for navigation, such as global positioning system (GPS) receivers or inertial measurement units (IMUs).

#### 2.2.1. Traditional Methods

The first attempts for aerial fire detection began around 1920 when planes were used for forest fire detection as a result of their unsuccessful deployment for the extinguishing of fires [70]. In 1986, Stearns et al. [71] captured IR images by the Flying Infrared Signatures Technology Aircraft of the U.S. Air Force Geophysics Laboratory and described a spatial and spectral analysis methodology to provide wildfire detection and monitoring for fire control. Similarly, Den Breejen et al. [72] used a single manned aerial vehicle for forest fire detection and tracking; however, although the operation of manned aerial vehicles is safer in busy airspace around fire [12], these vehicles are typically large and require increased operational costs making them a less useful tool for fire detection.

In recent times, the deployment of UAVs is considered to be a better option for the task of forest fire detection. More specifically, to achieve forest fire detection and tracking, Yuan et al. [73] used median filtering for noise reduction, color analysis based on CIELAB color space, Otsu threshold for fire segmentation, and blob counting. More recently, in [74] they used visual images captured by an optical camera of an unmanned aerial vehicle. Then, two color spaces, namely, RGB and HIS were chosen as inputs of a fuzzy logic rule and an extended Kalman filter was employed to adapt environmental condition variations and to perform smoke detection. Extending the color-based methods in [75,76] fire flame and smoke pixels are segmented using both color and motion characteristics. For the estimation of color features, they utilized three color spaces RGB, YCbCr, and HIS, whereas for the extraction of motion characteristics they noted that flames have turbulent movement or disordered characteristics. Thus, an optical flow algorithm was used to examine the motion characteristic of forest fires and extract fire motion pixels using dynamic background analysis.

Aiming to identify the fire location in terms of latitude, longitude, and altitude in [77], the authors used a DJI F550 hexacopter and applied two coordinate system transformations between the body-fixed frame, namely north-east-down frame (NED) and Earth-centered Earth-fixed (ECEF) frame. Then, a rule-based color model combining RGB and YCbCr color spaces was used for the identification of fire pixels. A fire simulation platform based on the Unity game engine and robot operating systems (ROS) was developed by Esfahlani [78]. The video data were collected through a monocular camera and navigation relied on a simultaneous localization and mapping (SLAM) system. A fire detection algorithm based on color, movement attributes, temporal variation of fire intensity, and its accumulation around a point was deployed. Finally, a mixed reality (MR) system incorporating physical and virtual elements was adopted to visualize and test the developed system.

Sudhakar et al. [79] proposed a method for forest fire detection through UAVs equipped with an optical and an infrared camera. They used a LAB color model and a motion-based algorithm followed by a maximally stable extremal regions (MSERs) extraction module. For improved presentation, the extracted forest fire detections are joined with landscape information and meteorological data. In [80] two types of UAVs, a fixed-wing drone and a rotary-wing drone equipped with optical and thermal cameras were used. As soon as the fixed-wing drone detects a fire, the rotary-wing drone will fly at a much lower altitude (10 to 350 m) compared to a fixed-wing UAV (350 to 5500 m), thus having better and more detailed visibility of the area and reducing false alarms through a neural network. Chen et al. [81] used optical and infrared sensors and data to train a CNN first for smoke detection and then for flame detection. In [82], the authors developed a system consisting of a central station and several aerial vehicles equipped with infrared or visual cameras, aiming to increase the coverage area. For fire detection, they applied a threshold for fire segmentation and then performed color and fire contour analysis.

#### 2.2.2. Deep Learning Methods

Zhao et al. [83] used a UAV equipped with GPS and deployed a saliency detection algorithm for localization and segmentation of the fire area in aerial images. Then, a 15 layered deep convolutional neural network architecture was employed for both low and high-level fire feature extraction and classification. Tang et al. [84] captured 4K data using a ZenMuse XT2 dual vision sensor and applied an adaptive sub-region select block to detect fire candidate areas in 4K resolution images. Then, a YOLOv3 backbone architecture was used for fire detection. Jiao et al. [85] used a UAV to capture and transmit images to the ground station in real-time deploying a YOLOv3 network for fire detection. Furthermore, they deployed a UAV equipped with a visible and an infrared camera for image acquisition [86]. The onboard computer carried by UAV can perform local image processing and mission planning through a YOLOv3-tiny architecture. In addition, a ground station receives images and location information of fire spots, contributing to the detection of forest fires. Furthermore, it provides operational commands to the UAV for path planning and re-planning. Integrating fog computing and CNNs, Srinivas and Dua [87] employed a UAV to reduce the false alarm rates towards the task of forest fire detection at an early stage. More recently, Barmpoutis, et al. [88,89] used an optical 360-degree complementary metal-oxide-semiconductor (CMOS) camera mounted on a UAV to capture an unlimited field of view images. More specifically, they converted the equirectangular raw data to cubemap and stereographic projections, respectively. Then, they used deep neural networks and exploited fire dynamic textures aiming to reduce false alarms that are caused due to clouds, sunlight reflections, and fire/smoke-colored objects. Experimental results demonstrate the great potential of the proposed system for both flame and smoke detection.

### 2.3. Spaceborne (Satellite) Systems

Recently, mainly due to the large number of satellites launched and the decrease of associated costs, there are many research efforts to detect forest wildfires from satellite images. Specifically, a set of satellites were designed for Earth observation (EO, e.g., environmental monitoring or meteorology). Depending on their orbit, satellites can be broadly classified into various categories, each having their advantages and disadvantages. The most important categories include: (a) the geostationary orbit (GEO), which is a circular orbit with an altitude of 35,786 kilometers and zero inclination, so that the satellite does not move at all relative to the ground, providing a constant view of the same surface area, (b) the low Earth orbit (LEO), which has an altitude of 2000 km or less, requires the lowest amount of energy for satellite placement and provides high bandwidth and low communication latency, (c) the polar sun-synchronous orbit (SSO), which is a nearly polar orbit that passes the equator at the same local time on every pass. Most EO satellites are in specific low Earth polar SSO orbits, whose altitude and inclination are precisely calculated so that the satellite will always observe the same scene with the same angle of illumination coming from the Sun, so shadows appear the same on every pass.

Data from sun-synchronous satellites have high spatial resolution but low temporal resolution, whereas geostationary satellites have high temporal resolution but low spatial resolution. Some satellites of the first category, like Landsat or Sentinel satellites, have a large revisit time (eight-day repeat cycle for LandSat-7/8 and approximately 2–3 days at mid-latitudes for Sentinel 2A/2B). Hence, they are unsuitable for real-time active forest fire detection, but only for less time-sensitive tasks, e.g., burnt area estimation, and therefore their studies fall within the scope of this paper.

#### 2.3.1. Fire and Smoke Detection from Sun-Synchronous Satellites

Imaging sensors in sun-synchronous satellites include three multispectral imaging sensors, namely advanced very-high-resolution radiometer (AVHRR) [90], moderate resolution imaging spectroradiometer (MODIS) [91], and visible infrared imaging radiometer suite (VIIRS) [92,93], whose data have also been used for wildfire detection. The advanced very-high-resolution radiometer (AVHRR/3) is a multipurpose imaging instrument that measures the reflectance of the Earth and has been used for global monitoring of cloud cover, sea surface temperature, ice, snow, and vegetation cover characteristics [90]. AVHRR instruments are or have been carried by the National Oceanic and Atmospheric Administration (NOAA) family of polar-orbiting platforms (polar-orbiting operational environmental satellite—POES) and European Meteorological Operational (MetOp) satellites. The instrument provides six channels, three in the visible/near-infrared region and three thermal infrared channels, with 1 km spatial resolution. The moderate resolution imaging spectroradiometer (MODIS), onboard the National Aeronautics Space Administration (NASA) EO Terra and Aqua satellites that have a revisit time of 1–2 days, capture data in 36 spectral bands ranging in wavelengths from 0.4 to 14.4 μm and at varying spatial resolutions (2 bands at 250 m, 5 bands at 500 m, and 29 bands at 1 km). MODIS was succeeded by the visible infrared imaging radiometer suite (VIIRS) instrument onboard the Suomi National Polar-orbiting Partnership (NPP) and NOAA-20 weather satellites. The instrument provides 22 different spectral bands, i.e., 16 moderate-resolution bands (M-bands, 750 m), 5 imaging resolution bands (I-bands, 375 m), and 1 day/night panchromatic band (750 m).

##### Traditional Methods

These imaging sensors have also been extensively applied for near-real-time wildfire detection. For instance in [94], Sayad et al. combined big data, remote sensing, and data mining algorithms (artificial neural network and SVM) to process big data collected from MODIS images and extract insights from them to predict the occurrence of wildfires. More specifically, they used pre-processed MODIS data to create a dataset based on three parameters related to the state of the crops: namely normalized difference vegetation index (NDVI), land surface temperature (LST), and thermal anomalies. For wildfire prediction, they used two different supervised classification approaches based on neural networks and SVM, achieving good prediction accuracies, i.e., 98.32% and 97.48%, respectively. Results were assessed using several validation strategies (e.g., classification metrics, cross-validation, and regularization) and comparisons with other wildfire prediction models, demonstrating the efficiency of the model in predicting the occurrence of wildfires.

Several papers deal with the problem of smoke detection based on MODIS data, which is a very challenging problem given its strong similarity with clouds, haze, and other similar phenomena. Shukla et al. [95] proposed an algorithm for automatic detection of smoke using MODIS data, which was based on a multiband thresholding technique for discriminating between smoke plumes and clouds. Results suggested that the algorithm was able to isolate smoke pixels in the presence of other scene types, such as clouds, although it performed better in identifying fresh dense smoke as compared to highly diffused smoke. Similarly, Li et al. [96] proposed an approach to automatically separate smoke plumes from clouds and background by analyzing MODIS data. Specifically, a previous approach proposed by Li et al. [97] for the AVHRR sensor was improved based on spectral analysis among the smoke, cloud, and underlying surface using MODIS data. Specifically, a multi-threshold method was used for extracting training sample sets to train a back-propagation neural network (BPNN) to discriminate between three classes: (smoke, cloud, and underlying surface). Results using MODIS data of several forest fires occurred in different places and different dates were satisfactory. Advantages include the ability of the algorithm to detect smoke plumes in different seasons using seasonal training data sets, as well as that it provides quantitative and continuous outputs of smoke and other objects.

Many researchers used active fire products derived from these sensors to assess various other proposed algorithms. Hally et al. [98] examined the performance of a threshold algorithm against commonly used products such as the VIIRS active fire product, to determine the completeness of anomaly capture. Specifically, the study considers two commonly used active fire products: the MODIS Collection 6 (MOD/MYD14) 1 km active fire product as outlined in Giglio et al. [99] and the VIIRS 375 m (VNP14IMG) active fire product described in Schroeder et al. [93]. In both cases, the geographic position of the detected hotspots, as well as the time of satellite overpass, were used. Also, Wickramasinghe et al. [100] compared the Advanced Himawari Imager—Fire Surveillance Algorithm (AHI-FSA) across the Northern Territory of Australia (1.4 million km^2^) over ten days with the well-established active fire products from satellites: MODIS and VIIRS.

Finally, the Chinese HuanJing sun-synchronous satellites (“HuanJing” means “environment” in Chinese) are satellites for disaster and environmental monitoring that are capable of visible, infrared, multi-spectral, and synthetic aperture radar imaging. Lin et al. [101] presented a spatio–temporal model (STM) based forest fire detection method that uses multiple images of the inspected scene based on Huanjing-1B satellite images. A comparison of detection results demonstrated that the proposed algorithm is useful to represent the spatio–temporal information contained in multi-temporal remotely sensed data.

##### Deep Learning Methods

Deep Learning methods have also been recently applied for fire and smoke detection from multispectral satellite images. Ba et al. [102] presented a new large-scale satellite imagery dataset based on MODIS data, namely USTC_SmokeRS, consisting of 6225 satellite images from six classes (i.e., cloud, dust, haze, land, seaside, and smoke) and covering various areas/regions over the world. Using this dataset, they evaluated several state-of-the-art deep learning-based image classification models for smoke detection and proposed *SmokeNet*, a new CNN model that incorporated spatial- and channel-wise attention in CNN to enhance feature representation for scene classification. Also, Priya et al. [103] used a dataset of 534 RGB satellite images from different sources, including MODIS images from the NASA Worldview platform and Google. An effective approach using an Inception-v3 CNN framework and transfer learning was used for fire and non-fire image classification. Then, the fire regions were extracted based on thresholding and local binary patterns.

#### 2.3.2. Fire and Smoke Detection from Geostationary Satellites

Regarding satellite imagery from geostationary satellites, important work for fire and smoke detection has already been performed using the advanced Himawari imager (AHI) sensor of the Himawari-8 weather satellite. Himawari 8 is a new generation of Japanese geostationary weather satellites operated by the Japan Meteorological Agency. AHI-8 has significantly higher radiometric, spectral, and spatial resolution than its predecessor.

Regarding Europe and the US, two additional sensors that are installed in geostationary satellites are the European Space Agency (ESA) Meteosat second generation (MSG, a satellite series)-spinning enhanced visible and infrared imager (SEVIRI) sensor and the NASA geostationary operational environmental satellite (GOES)-16 advanced baseline imager (ABI) sensor. The MSG-SEVIRI geostationary sensor is a 50 cm diameter aperture, line-by-line scanning radiometer, which provides image data in 12 spectral channels (four visible and near-infrared (NIR), including a broadband high resolution (1 km) visible channel, and eight thermal IR with a resolution of 3 km) with a baseline repeat cycle of 15 min. GOES-16 is the first of the GOES-R series of the geostationary operational environmental satellite (GOES) operated by NASA and the NOAA. The advanced baseline imager (ABI) is its primary instrument, providing high spatial and temporal resolution imagery of the Earth through 16 spectral bands at visible and infrared wavelengths.

##### Traditional Methods

Hally et al. [98] extended previous work by the same authors Hally et al. [104] using AHI sensor data from the Himawari geostationary satellite in the application of a multi-temporal method of background temperature estimation, known as the broad area training (BAT). This method involves a two-step process for geostationary data: a preprocessing step, where AHI Band 7 images are aggregated and then a fitting step, where this spatially aggregated data are used in individual pixel fitting using a single value decomposition (SVD) process. These fittings at the pixel level can then be compared to the raw brightness temperature data as measured by the satellite sensor, to identify thermal anomalies such as those caused by an active fire. Results are seen to compare favorably to active fire products produced by low Earth orbit satellite data during the period of study. Fatkhuroyan et al. [105] perform a study of data from fires in Sumatera and Kalimantan regions in August, September, and October 2015 and concluded that smoke detection and monitoring is feasible using pseudo-RGB images consisting of one visual channel and two near-infrared channels. A limitation revealed by this study is that Himawari-8/AHI is a passive sensor that very dependent on the reflection of solar radiance, so it can only monitor the forest fire during the day-time. Xu et al. [106] investigated the feasibility of extracting real-time information about the spatial extents of wildfires using the Himawari-8 satellite. The algorithm is based on previous work using the MODIS sensor: it first identifies possible hotspots and then eliminates false alarms by applying certain thresholds, similar to Giglio et al. [99]. False alarms are then rejected by cloud, water, and coast tests based on the additional bands and comparison with neighboring pixels. Results demonstrated that fire detection is robust to smoke and moderate cloud obscuration and sensitive enough for early detection of wildfires.

Typically, only temporal-based fire detection algorithms are used for geostationary orbital sensors, detecting the fire by analyzing multi-temporal changes of brightness temperature (BT). On the other hand, polar-orbiting platforms, use spatial-based fire detection algorithms, which are commonly classified either “fixed-threshold” or “contextual”. Aiming to combine the merits of both approaches, Xie et al. [107] presented a spatio–temporal contextual model (STCM) that fully exploits geostationary data’s spatial and temporal dimensions using data from Himawari-8 Satellite. They applied an improved robust fitting algorithm to model each pixel’s diurnal temperature cycles (DTC) in the middle and long infrared bands. For each pixel, a Kalman filter was used to blend the DTC to estimate the true background brightness temperature.

Significant research results have also been produced using data from the GOES ABI and MSG SEVIRI instruments. A multi-temporal change-detection technique, namely robust satellite techniques for fires detection and monitoring (RST-FIRES) using data from the MSG-SEVIRI sensor. Filizzola et al. [108] was seen to be very efficient for the timely detection of even small/short-living fire incidents. Furthermore, Di Biase et al. [109] updated the satellite fire detection (SFIDE) algorithm, previously proposed by the same authors Laneve et al. [110] to reduce the false alarm rate. Specifically, they improved the estimation of the reference temperature used to define a fire pixel, the cloud mask accuracy, and the exploitation of the high refresh rate of the images to implement several tests for more accurate detection of forest fires. In Hall et al. [111], both satellite imaging sensors (GOES ABI and MSG SEVIRI) were found to be very efficient in detecting active fire incidents. Additional sensing systems providing such broad spatial coverage and favoring improved geostationary satellite fire data consistency across regions could further improve performance. Reference fire data were derived from Landsat-8 Operational Land Imager (OLI) 30 m imagery using the Schroeder et al. [112] Landsat-8 OLI automated fire detection algorithm.

##### Deep Learning Methods

Very recently, Larsen et al. [113] presented a deep FCN for predicting fire smoke in satellite imagery in near-real-time (NRT) using data from the Himawari-8/AHI sensor. Also, Phan et al. [114] proposed a novel remote wildfire detection framework based on GOES-16 sensor imagery, which contains three distinct stages: (i) a streaming data processing component to purify and examine raw image data for ROI’s, (ii) an early wildfire prediction component using deep learning architectures to grab spatial and spectral designs for more accurate and robust detection, and (iii) a streaming data visualization dashboard for potential wildfire incidents.

#### 2.3.3. Fire and Smoke Detection Using CubeSats

A recent trend in remote sensing from satellites is using “Cubsats”, i.e., miniaturized satellites that typically weigh between 1 and 10 kg and follow the popular ‘CubeSat’ standard [115], which defines the outer dimensions of the satellite within multiple cubic units of 10 × 10 × 10 cm and can accommodate small technology payloads to perform different scientific research or commercial functions and explore new space technologies. Technically, it is easier for CubeSats to function in the LEO-zone due to their unique characteristics. More than 1100 CubeSats have been successfully launched by several universities and companies worldwide, as of January 2020.

The latest advances in satellite imagery had allowed CubeSats to rapidly discover wildfires. Barschke et al. (2017) [116] described a nanosatellite called TUBIN (Technische Universität Berlin Infrared Nanosatellite), which was designed to validate an infrared microbolometer for wildfire remote sensing. Another constellation of four 6U Cubesats for monitoring forest fires and other natural disasters was proposed in Africa and Asia [117]. The payload is an optical sensor with three spectral bands (green, red, and near-infrared) with a revisit time of 72 h. Shah et al. [118] proposed a system consisting of a constellation of nanosatellites equipped with multi-spectral visible to Infrared (IR) cameras and a ground station, which will allow all surface points on the planet to be revisited at least once in an hour. Capturing a surface location with high resolution in MWIR and LWIR allows for the precise estimation of the thermal output of the surface. Simulations indicated that a fire of about four hundred square meters can be detected using a payload of a multispectral IR camera measuring incident power in two thermal infrared bands (mid-wave and long-wave). The system will use onboard data processing, enabling an early wildfire warning within 30 min and minimizing bandwidth requirements. Additionally, compressed raw images can be transmitted to the ground station to provide global thermal data updated every 90 min. The first satellite is planned for launch in late 2020 with the data available for research purposes.

## 3. Discussion and Conclusions

Three categories of early fire and smoke detection systems have been analyzed and compared (Table 1) thoroughly in this literature review paper, namely terrestrial, unmanned aerial vehicles, and satellite-based systems. In general, terrestrial systems tend to be more efficient in terms of accuracy and response time to wildfire incidents. Furthermore, these systems offer high spatial resolution depending on the camera resolution and the viewing angle/distance; however, their coverage is limited when compared to the other two solutions, as the cameras are placed in fixed positions and additional limitations may apply (e.g., occlusions).

On the other hand, aerial-based systems gained recently a lot of attention due to the rapid development of UAV technology. Such systems provide a broader and more accurate perception of the fire, even in regions that are inaccessible or considered too dangerous for fire-fighting crews. In addition, UAVs can cover wider areas and are flexible, in the sense that they monitor different areas, as needed. Recent technological achievements have led to better camera resolutions offering high spatial resolution, wider field of view, and better battery autonomy. The latest UAVs are equipped with both visible and infrared cameras, improving the detection accuracy and allowing night operation; however, they are affected by weather conditions and, in many cases, their flight time is limited.

Finally, Earth Observation satellite systems have been used successfully for wildfire detection, mainly due to their large-scale coverage. The majority of satellites providing earth imagery are either geostatic or in the near-polar sun-synchronous orbit and include multispectral imaging sensors. Sun-synchronous satellites provide data with high spatial resolution but low temporal resolution, while geostationary satellites have a high temporal resolution but low spatial resolution. More recently, advances in nanomaterials and micro-electronics technologies have allowed the use of tiny low-Earth-orbiting satellites, known as CubeSats. CubeSats have significant advantages in comparison with traditional satellites regarding smoke and fire detection, since they are more effective in terms of costs, temporal resolution/response time, and coverage. In addition, they are smaller in size than traditional satellites and need less time to be put into orbit; however, one issue that needs to be tackled is their poor ability to transmit large amounts of data to the ground. From the aforementioned analysis, it is clear that each category has its advantages and disadvantages. To this end, recent research efforts on wildfire detection [127,128,129] focus either on the combination of these technologies or on the use of additional input sources such as crowdsourcing, social media, and weather forecasting.

It is worth mentioning that most of the institutions and agencies aiming to support wildfire management at the national and regional level use either satellites or combine them with a small fleet of planes to detect and map the extent, spread, and impact of forest fires [130,131]. Furthermore, various organizations have installed network-connected optical cameras in or near wildland areas sharing live images on the web to assist early forest fire detection [132].

**Figure 3:**
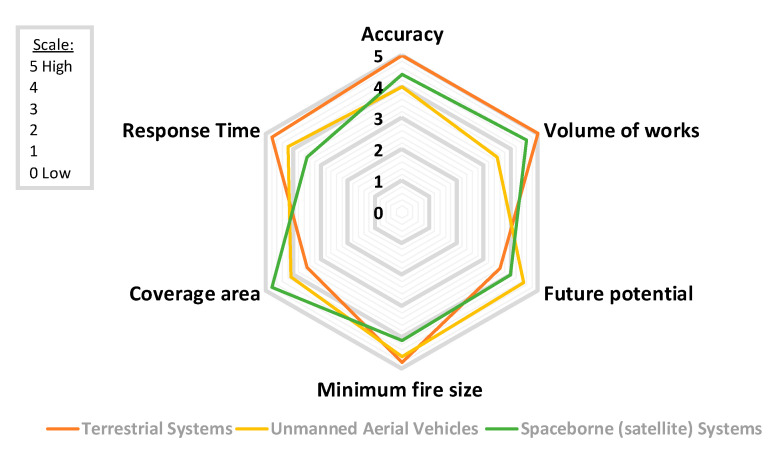
Radar chart showcasing the findings of this review for different early forest fire detection systems with regards to accuracy, response time, coverage area, future potential, and volume of works in the scale 0 (low) to 5 (high).

A detailed comparison between the three categories of early fire detection systems in terms of the performance (Accuracy), number of research papers (Volume of works), the potential for future improvement (Future potential), the minimum fire size that can be detected (Minimum fire size), the monitoring area covered by the system (Coverage area), and time needed for early fire detection (Response time) in the scale 0 (low) to 5 (high) is shown in Figure 3. Most of the literature shows that terrestrial systems have been extensively studied, achieving high accuracy rates and fast response times, despite the limited coverage area that they offer. Thus, large networks of ground sensors can be deployed to increase the coverage area; however, in this case, a trade-off between the number of sensors, cost, and complexity is required. On the other hand, aerial and satellite-based systems provide better coverage. These systems have already shown their great potential and accuracy rates and response times.

Also, the terrestrial and aerial-based systems can detect fires at a very early stage depending on their distance from the fire and their spatial resolution in parallel with short latency time [132]. In contrast, regarding satellite-based systems, time latency and minimum detectable fire size are expected to be improved in the following years. Currently, imaging sensors in sun-synchronous-orbiting satellites, such as MODIS, can detect after observation very small fires (up to 50 m^2^) under near-ideal conditions and an average size of 30 × 30 = 900 m^2^ under a variety of conditions [133]. Regarding the latency time, MODIS fire products are produced and delivered to fire managers partners in near-real-time (within 2–4 h of when MODIS collected the observations) [133]. On the other hand, geostationary sensors, like Himawari-8 AHI, can provide observations every 10–30 min, making them ideal for near-real-time fire surveillance, at the cost of a higher spatial resolution (2 km pixel size, which can be reduced to 500 m) [100]. Furthermore, the creation of new datasets for the training of novel deep learning algorithms (e.g., super-resolution), as well as advances in transmission technologies will further contribute towards this direction.

**Figure 4:**
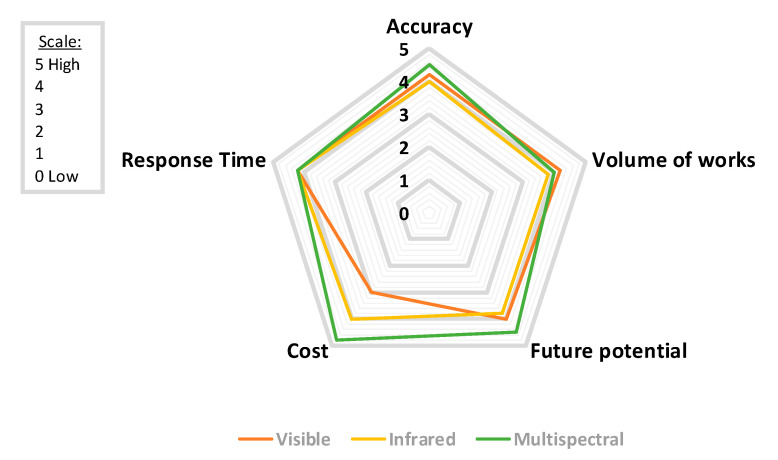
Radar chart showcasing the findings of this review for different sensor types with regards to accuracy, response time, cost, future potential, and volume of works in the scale 0 (low) to 5 (high).

Similarly, Figure 4 demonstrates some attributes for each of the three sensor types: visible, infrared, and multispectral. Although the research community has thoroughly utilized optical-based systems, multispectral approaches seem to achieve better accuracy rates, due to the complementarity of information provided by different spectral bands; however, the use of multispectral technology increases significantly the overall cost of the system. This fact justifies the extensive use of low-cost optical sensors by many research works in the literature. Nevertheless, the wider use of multispectral sensors in different systems is expected to further improve the performance of early wildfire detection systems. To this end, extensive research is still needed on systems that integrate multimodal sensing technologies along with advanced deep-learning algorithms.

**Figure 5:**
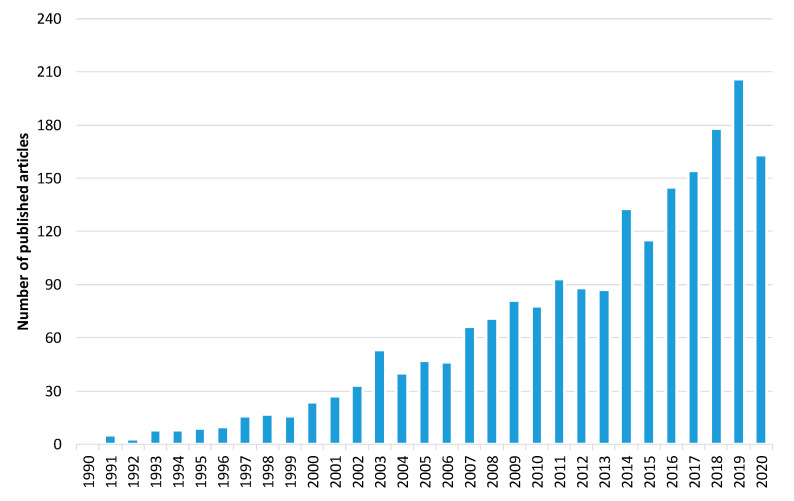
The number of published articles per year related to forest fire detection. Data retrieved from Web of Science [134] for dates between 1990 to October 2020.

**Figure 6:**
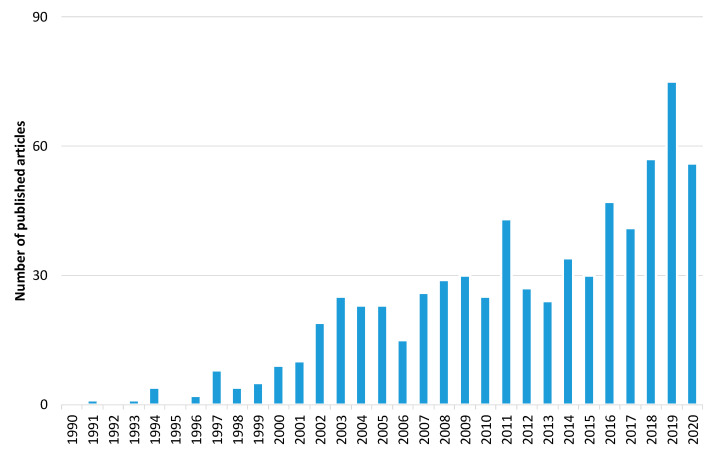
The number of published articles per year related to forest fire detection in the imaging research area. Data retrieved from Web of Science [134] for dates between 1990 to October 2020.

**Figure 7:**
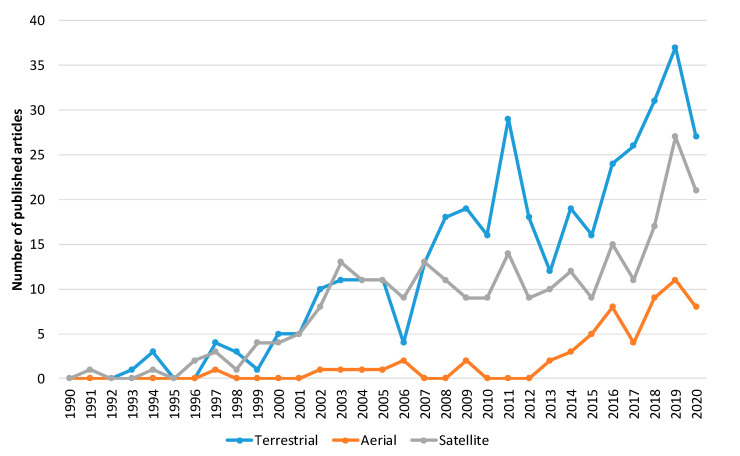
The number of published articles per year for terrestrial, aerial, and satellite-based systems. The analysis was performed for forest fire detection in the imaging research area. Data retrieved from Web of Science [134] for dates between 1990 to October 2020.

Furthermore, to explore the evolution of the forest fire detection research domain, we carried out a bibliometric analysis. The initial search yielded over 2024 papers related to forest fire detection published in the Web of Science (WoS) database [134]. Figure 5 shows the trend in the number of articles published between 1 January 1990 and 31 October 2020. Narrowing the results to only the imaging research area, the search yielded 697 published articles (Figure 6). These results show that there is a growth in publications in the last 30 years. Of these, 378 are related to forest fire detection based on terrestrial systems, 59 based on aerial systems, and 260 based on satellite systems (Figure 7). The results of Figure 7 indicate that the field of forest fire detection in the image research area for terrestrial, aerial, and satellite-based systems is still evolving.

**Figure 8:**
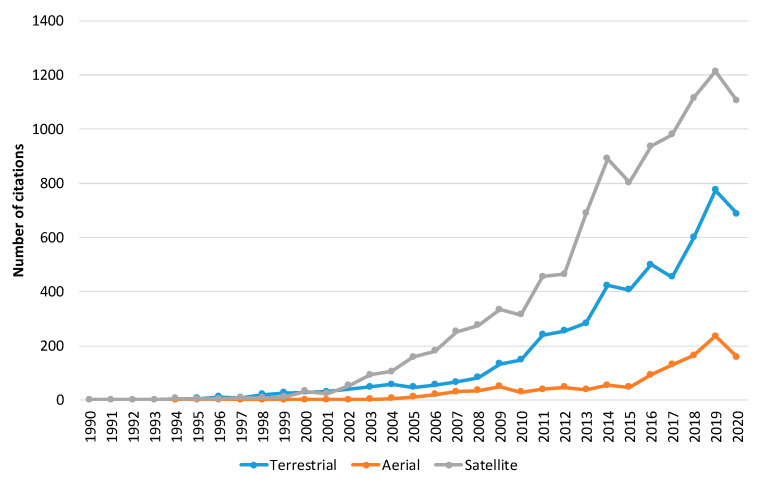
The number of times cited the published articles per year for terrestrial, aerial, and satellite-based systems. The analysis was performed for forest fire detection in the imaging research area. Data retrieved from Web of Science [134] for dates between 1990 to October 2020.

**Figure 9:**
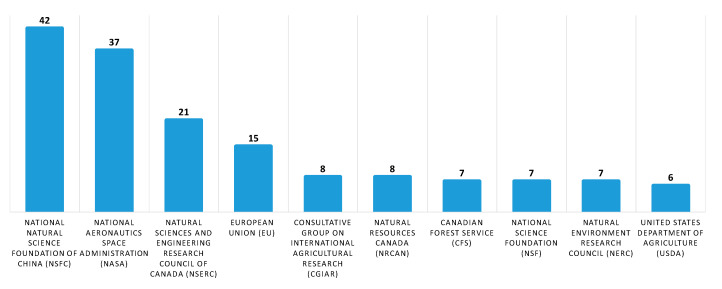
Organizations and agencies that funded most of the published articles for forest fire detection in the imaging research area. Data retrieved from Web of Science [134] for dates between 1990 to October 2020.

**Figure 10:**
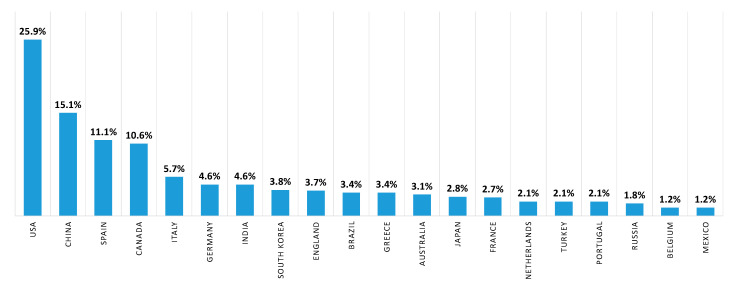
Authors’ affiliation by country (%) for forest fire detection in the imaging research area. Data retrieved from Web of Science [134] for dates between 1990 to October 2020.

Subsequently, to provide information on the identity of papers and the corresponding systems that receive the most citations, we performed a quantitative citation analysis. A citation occurs when one paper refers to another paper. In this analysis (Figure 8), we identified that papers related to forest fire detection in the imaging satellite-based research area receive the most citations followed by terrestrial and aerial-based papers. Finally, to identify the study area of the aforementioned papers, a study analysis of the funding agencies was performed. In Figure 9, ten of the organizations and agencies that were the major sponsors of these papers are shown. More specifically, the National Natural Science Foundation of China (NSFC) has funded 42 papers related to forest fire detection in the imaging research area, while National Aeronautics Space Administration (NASA) has funded 37 papers. In addition, organizations and agencies from Canada, the European Union, France, the USA, and the UK have funded more than 79 papers related to the field of imaging-based forest fire detection. Similarly, in the second analysis, the authors’ countries of affiliation were mapped (Figure 10). To this end, over 25 percent and 15 percent of the authors are affiliated with an organization that is based in the USA and China, respectively.

## Figures and Tables

**Table 1:** Multispectral imaging systems and their characteristics.

	(Satellite)-Sensor	Spectral Bands	Access to the Data	Specs/Advantages/Limitations	Spatial Scale	Spatial Resolution	Data Coverage	Accuracy Range
**Terrestrial**	**Optical**	Visible spectrum	Both web cameras and image and video datasets are available	Easy to operate, limited field of view, need to be carefully placed in order to ensure adequate visibility.	Local	Very high spatial resolution (centimeters) depending on camera resolution and distance between the camera and the event	Limited coverage depending the specific task of each system	85%–100% [35,40,58,60]
	**IR**	Infrared spectrum						
	**Multimodal**	Multispectral						
**Aerial**	**Optical**	Visible spectrum	Limited number of accessible published data	Broader and more accurate perception of the fire, cover wider areas, flexible, affected by weather conditions, limited flight time.	Local—Regional	High spatial resolution depending on flight altitude, camera resolution and distance between the camera and the event	Coverage of hundred hectares depending on battery capacity.	70%–94.6% [75,86,89]
	**IR**	Infrared spectrum						
	**Multimodal**	Multispectral						
**Satellite**	**Terra/Aqua-MODIS** [118]	36 (0.4–14.4 μm)	Registration Required (NASA)	Easily accessible, limited spatial resolution, revisit time: 1–2 days	Global	0.25 km (bands 1–2) 0.5 km (bands 3–7) 1 km (bands 8–36)	Earth	92.75%–98.32% [94,95,96,99,102]
	**Himawari-8/9—AHI-8** [119]	16 (0.4–13.4 μm)	Registration Required/ (Himawari Cloud)	Imaging sensors with high radiometric, spectral, and temporal resolution. 10 min (Full disk), revisit time: 5 min for areas in Japan/Australia)	Regional	0.5 km or 1 km for visible and near-infrared bands and 2 km for infrared bands	East Asia and Western Pacific	75%–99.5% [98,100,104,105,106,107,113]
	**MSG—SEVIRI [120]**	12 (0.4–13.4 μm)	Registration Required (EUMETSAT	Low noise in the long-wave IR channels, tracking of dust storms in near-real-time, susceptibility of the larger field of view to contamination by cloud and lack of dual-view capability, revisit time: 5–15 min	Regional	1 km for the high-resolution visible channel 3 km for the infrared and the 3 other visible channels	Atlantic Ocean, Europe and Africa	71.1%–98% [108,109,110,111]
	**GOES-16ABI** [121]	16 (0.4–13.4 μm)	Registration Required (NOAA)	Infrared resolutions allow the detection of much smaller wildland fires with high temporal resolution but relatively low spatial resolution, and delays in data delivery, revisit time: 5–15 min	Regional	0.5 km for the 0.64 μm visible channel 1 km for other visible/near-IR 2 km for bands > 2 μm	Western Hemisphere (North and South America)	94%–98% [111,114]
	**HuanJing (HJ)-1B—WVC** (Wide View CCD Camera)/**IRMSS** (Infrared Multispectral Scanner) [122]	WVC: 4 (0.43–0.9 μm) IRMSS: 4 (0.75–12.5 μm)	Registration Required	Lack of an onboard calibration system to track HJ-1 sensors’ on-orbit behavior throughout the life of the mission, revisit time: 4 days	Regional	WVC: 30 m IRMSS: 150–300 m	Asian and Pacific Region	94.45% [101]
	**POES/MetOp—AVHRR** [123]	6 (0.58–12.5 μm)	Registration Required (NOAA)	Coarse spatial resolution, revisit time: 6 h	Global	1.1 km by 4 km at nadir	Earth	99.6% [97]
	**S-NPP/NOAA-20/NOAA—VIIRS-375 m** [124,125]	16 M-bands (0.4–12.5 μm) 5 I-bands (0.6–12.4 μm) 1 DNB (0.5–0.9 µm)	Registration Required (NASA)	Increased spatial resolution, improved mapping of large fire perimeters, revisit time: 12 h	Global	0.75 km (M-bands) 0.375 km (I-bands) 0.75 km (DNB)	Earth	89%–98.8% [93]
	**CubeSats** (data refer to a specific design from [126])	2: MWIR (3–5 μm) and LWIR (8–12 μm)	Commercial access planned	Small physical size, reduced cost, improved temporal resolution/response time, Revisit time: less than 1 h.	Global	0.2 km	Wide coverage in orbit	The first satellite is planned for launch in late 2020

## Abbreviations

ABI	Advanced Baseline Imager
ADF	Adaptive Decision Fusion
AHI	Advanced Himawari Imager
AVHRR	Advanced Very-High-Resolution Radiometer
BAT	Broad Area Training
BPNN	Back-Propagation Neural Network
BT	Brightness Temperature
CCD	Charge-Coupled Device
CMOS	Complementary Metal-Oxide-Semiconductor
CNN	Convolutional Neural Network
DC	Deep Convolutional
DL	Deep Learning
DTC	Diurnal Temperature Cycles
ECEF	Earth-Centered Earth-Fixed
EO	Earth Observation
ESA	European Space Agency
FCN	Fully Convolutional Network
GAN	Generative Adversarial Network
GEO	Geostationary Orbit
GOES	Geostationary Operational Environmental Satellite
GPS	Global Positioning System
h-LDS	higher-order LDS
HMM	Hidden Markov Models
HOF	Histograms of Optical Flow
HOG	Histograms of Oriented Gradients
HoGP	Histograms of Grassmannian Points
IMU	Inertial Measurement Unit
IoMT	Internet of Multimedia Things
IR	InfraRed
LDS	Linear Dynamical Systems
LEO	Low Earth Orbit
LST	Land Surface Temperature
LSTM	Long Short-Term Memory
LWIR	Long Wavelength InfraRed
MetOp	Meteorological Operational
MODIS	Moderate Resolution Imaging Spectroradiometer
MR	Mixed Reality
MSER	Maximally Stable Extremal Regions
MSG	Meteosat Second Generation
MWIR	Middle Wavelength InfraRed
NDVI	Normalized Difference Vegetation Index
NED	North-East-Down
NIR	Near-InfraRed
NOAA	National Oceanic and Atmospheric Administration
NPP	National Polar-orbiting Partnership
NRT	Near-Real-Time
OLI	Operational Land Imager
PISA	Pixelwise Image Saliency Aggregating
POES	Polar-orbiting Operational Environmental Satellite
R-CNN	Region-Based Convolutional Neural Networks
ReLU	Rectified Linear Unit
RST	Robust Satellite Techniques
SEVIRI	Spinning Enhanced Visible and Infrared Imager
SFIDE	Satellite Fire Detection
sh-LDS	stabilized h-LDS
SLAM	Simultaneous Localization and Mapping
SroFs	Suspected Regions of Fire
SSO	Sun-Synchronous Orbit
STCM	Spatio–temporal Contextual Model
STM	Spatio–Temporal Model
SVD	Single Value Decomposition
SVM	Support Vector Machine
UAV	Unmanned Aerial Vehicles
VIIRS	Visible Infrared Imaging Radiometer Suite
WoS	Web of Science
WSN	Wireless Sensor Network
